# Expanded terminal sedation in end-of-life care

**DOI:** 10.1136/jme-2022-108511

**Published:** 2022-12-21

**Authors:** Laura Gilbertson, Julian Savulescu, Justin Oakley, Dominic Wilkinson

**Affiliations:** 1 Faculty of Medicine, Nursing and Health Sciences, Monash University, Clayton, Victoria, Australia; 2 Oxford Uehiro Centre for Practical Ethics, University of Oxford, Oxford, UK; 3 Centre for Biomedical Ethics, National University of Singapore Yong Loo Lin School of Medicine, Singapore; 4 Murdoch Children's Research Institute, Parkville, Victoria, Australia; 5 Monash Bioethics Centre, Monash University, Clayton, Victoria, Australia; 6 Newborn Care, John Radcliffe Hospital, Oxford, UK

**Keywords:** Euthanasia, Pain Management, Palliative Care, Right to Die, Terminal Care

## Abstract

Despite advances in palliative care, some patients still suffer significantly at the end of life. Terminal Sedation (TS) refers to the use of sedatives in dying patients until the point of death. The following limits are commonly applied: (1) symptoms should be refractory, (2) sedatives should be administered proportionally to symptoms and (3) the patient should be imminently dying. The term ‘Expanded TS’ (ETS) can be used to describe the use of sedation at the end of life outside one or more of these limits.

In this paper, we explore and defend ETS, focusing on jurisdictions where assisted dying is lawful. We argue that ETS is morally permissible: (1) in cases of non-refractory suffering where earlier treatments are likely to fail, (2) where gradual sedation is likely to be ineffective or where unconsciousness is a clinically desirable outcome, (3) where the patient meets all criteria for assisted dying or (4) where the patient has greater than 2 weeks to live, is suffering intolerably, and sedation is considered to be the next best treatment option for their suffering.

While remaining two distinct practices, there is scope for some convergence between the criteria for assisted dying and the criteria for ETS. Dying patients who are currently ineligible for TS, or even assisted dying, should not be left to suffer. ETS provides one means to bridge this gap.

## Clinical case

Mrs Johnson^1^ is a 35-year-old female patient who was recently diagnosed with advanced glioblastoma multiforme. Treatment has been deemed futile and she has been told by her medical team that she has less than 6 months to live. Her current main symptoms include headaches, weakness in her upper and lower limbs, as well as worsening loss of function, requiring assistance to open her bowels. Mrs Johnson is suffering from severe existential angst; she is fearful of death and is constantly teary, making it difficult for her to have any positive or meaningful experiences. She has support from a palliative care team and is receiving cognitive behavioural therapy with minimal effect. She describes her current state as ‘torture’ and asks to be sedated so that she will not experience any more suffering. Her partner is supportive of this decision. Notwithstanding her repeated requests, the medical team advises Mrs Johnson that they do not believe this would be appropriate. According to professional guidelines, sedation is reserved for dying patients who have less than 2 weeks to live. As a consequence, Mrs Johnson will likely continue to experience significant suffering until she is perceived to be closer to death.

## Introduction

Terminal sedation (TS) refers to the use of sedation in the terminally ill.^1^ It is a common practice, with approximately 12%–18% of dying patients worldwide receiving continuous sedation until the point of death.^2^ The terms ‘TS’ and ‘palliative sedation’ are often used interchangeably in the literature. In line with van Delden, we prefer ‘TS’ because it indicates that ‘an end-of-life decision is involved’.^3^ We do, however, acknowledge ‘palliative sedation’ as an alternative term to TS.

There are standard criteria for when TS is commonly regarded as acceptable. For example, de Graeff and Dean recommend that^1^:

1. Symptoms should be refractory (symptoms for which all reasonable treatment has failed).
2. The administration of sedatives should be proportionate to symptoms.
3. The patient should be imminently dying.

Where these criteria are met, TS is usually considered acceptable medical practice by ethicists, judiciaries and doctors alike.^1 4^ Contention remains, however, around the justifiable boundaries of TS and the acceptable expansion of its use. For example, is TS acceptable in cases where symptoms are not refractory, where sedatives are administered rapidly (ie, not proportionally), and/or where death is not imminent? In this paper, we use the term ‘expanded TS’ (ETS) to describe the use of sedation at the end of life which extends beyond the traditional limits.^2^

We will answer two key questions in this paper: first, is ETS morally permissible? Second, if so, in what circumstances is it morally permissible? To answer the first question, we will review existing literature on TS and challenges to the standard limits placed on its practice. To answer the second question, we will present arguments in favour of expanding TS beyond each of the individual limits mentioned above. We will specifically focus on jurisdictions where assisted dying is legal, since in such places there is acceptance of the permissibility of intentional hastening of death and existing criteria for this. As an example, we will refer to the assisted dying legislation in Victoria, Australia.

We will argue that patients have a right to access means of relief of suffering at the end of life, including ETS. Sedation should be accessible when it provides an appropriate means of relieving suffering at the end of life, even if the patient does not meet the traditional criteria for TS. This includes circumstances when earlier treatment options (eg, opioids) are likely to fail, when unconsciousness is a clinically desirable outcome, or when the patient meets the criteria for assisted dying.

We will further argue that ETS is a morally permissible form of relief of suffering for dying patients who are suffering but are currently ineligible for both TS and assisted dying. Importantly, ETS provides a means of relieving suffering for dying patients who are suffering intolerably but lack decision-making capacity. Although not the primary focus of this paper, there may be a further important role for ETS to treat end of life suffering in jurisdictions where assisted dying is not a lawful option.

## Ethical frameworks

In considering, broadly, whether ETS should be offered as part of palliative care, we might consider both negative and positive claims. For the former, we should assess whether existing ethical rules prohibit this practice. For the latter, we should consider if there are strong positive reasons that this practice should be offered. There are a range of different ethical approaches that might be applied. For example, a consequentialist or utilitarian approach would likely endorse the expansion of TS where the benefits (reduction of distress) would outweigh harms. In this paper, we will draw on two deontological principles that we suggest have wide appeal and relevance.

### Doctrine of double effect

One of the most widely cited ethical norms applied to end-of-life care is the doctrine of double effect (DDE). First introduced by Thomas Aquinas in Summa Theologica as a means to justify self-defence,^5^ the key idea of the DDE is that, where an action has two outcomes, one ‘bad’ and one ‘good’, the action is permissible where the actor intends the good outcome and merely foresees the bad.^5^

There are four traditional conditions required for the use of the DDE.^5^ McIntyre added a fifth condition.^5^ Taken together these conditions are that:

1. The action must be morally good (or at least not morally bad).
2. The actor must not intend the bad effect and if there is another way to achieve the good effect without the bad effect this should be pursued (ie, the action is a ‘last resort’).
3. The bad effect cannot be a means to achieving the good effect.
4. The good effect outweighs the bad.
5. The actor seeks to minimise the amount of harm in the bad effect.

Applied to conventional forms of TS, the DDE suggests that it is permissible to administer doses of sedatives at the end of life where the doctor intends to relieve suffering and merely foresees the risk of hastening their death, as long as this is proportionate to the patient’s symptoms and harms are minimised.^6^ More recent literature has considered the application of the DDE to a second potential ‘harm’ of TS—that of the removal of consciousness.^7 8^ Whether applied to the hastening of death or the removal of consciousness, the conditions of the DDE might provide the basis for the standard limiting criteria for TS.

One potential approach to justifying ETS would be to question the ethical relevance of the DDE. There is a wide body of literature criticising the DDE.^9 10^ If the DDE is rejected as a guiding principle, that would certainly widen the scope for TS. However, we have taken a different approach in this paper. The DDE continues to shape much of the ethical and legal literature concerning end-of-life care and TS and has been used to justify the traditional limits placed on TS. Below, we will explore the problems with drawing clear distinctions around intention. Ultimately, we argue that if the DDE is accepted as ethically significant for end-of-life care and TS, it nevertheless does not justify the traditional limits placed on TS. In the final section, we will consider positive ethical arguments in favour of the expansion of TS at the end of life, such as a right to relief of suffering.

### A right to relief of suffering at the end of life

If a practice is not prohibited, we need to assess whether there is a positive case for offering it. One basis for offering ETS would be a right to relief of suffering. This right arguably underlies much of modern palliative care.

A right to relief of suffering is a positive claim-right for patients to access treatment options to relieve their suffering at the end of life. It encompasses the following two principles: (1) dying patients should be entitled to access appropriate interventions to relieve their suffering at the end of life and (2) doctors have a duty to take reasonable steps to alleviate suffering in dying patients. This could include analgesia, sedation or even general anaesthesia^11^ where these would be effective at relieving distress.

A right to relief of suffering entails that dying patients should have access to clinically appropriate treatment options to manage their suffering; it does not give patients automatic access to all conceivable interventions. However, a right to relief of suffering might support the expansion of TS as a therapeutic option. Where a patient is suffering, and sedation would be a proportionate means of relieving that suffering, there would be a prima facie case for offering TS, even if this would fall outside one (or more) of the traditional limits.

## Is ETS morally permissible?

To determine whether the current limits to TS should remain, it will be useful to consider each of them in turn.

### Refractory symptoms

In the context of end-of-life care, ‘refractory’ refers to symptoms that persist despite aggressive palliative care measures to relieve them.^12^ It is sometimes claimed that, due to advancements in modern palliative care, very few patients suffer from refractory symptoms at the end of life.^13 14^ Consequently, TS is proposed as an option of last resort, reserved for terminally ill patients confronted with refractory and unbearable suffering.^15–17^

Arguments of last resort align with the second condition of the DDE: ‘if he could attain the good effect without the bad effect he should do so’.^5^ According to de Graeff and Dean, if unconsciousness is induced any earlier than is absolutely necessary, then it is plausible that the agent does in fact intend this effect.^1^

However, there may be patients for whom consciousness itself is a ‘bad’, and its intended removal could therefore be consistent with the DDE.^8^ In these cases, the principle of last resort does not hold. (The desirability of unconsciousness will be discussed further below where we consider symptoms which potentially render mere consciousness a ‘bad’ (or at least no longer a ‘good’)). Furthermore, even where the removal of consciousness and/or the potential hastening of death are deemed to be ‘bad’ effects, this does not necessarily mean that TS should be reserved as an option of last resort. First, the process of exhausting other palliative care measures can take time and involve significant distress along the way.^18^ Second, the assessment of how much suffering (and over what period of time) counts as ‘refractory’ is inherently subjective and value based. Arguably, it is only the patient who can ultimately determine when suffering becomes intolerable.^1 17^ The individual experiencing the symptom is best placed to weigh their current and future suffering against the foreseen harms of TS.^19^ An important implication of this argument is that, even where all standard palliative care options have not yet been tried, a dying patient might competently request (and be provided with) TS.

### Gradual sedation

A separate debate central to the practice of TS concerns the speed at which sedatives should be administered. The distinction between gradual and rapid administration was first introduced by Quill *et al*, who referred to ‘proportionate palliative sedation’ and ‘palliative sedation to unconsciousness’.^20^ Morita *et al* later use the terminology ‘gradual continuous deep sedation (CDS)’ and ‘rapid CDS’.^21^ In this paper, we will use ‘gradual TS’ (GTS) and ‘rapid TS’ (RTS) to represent the same concepts.

GTS involves the careful titration of sedatives for symptom relief which may, or may not, culminate in unconsciousness, while RTS involves the rapid administration of large doses of sedatives with the intention to sedate to unconsciousness.^20^ Most guidelines restrict the use of TS to the gradual form,^22^ on the basis of the principle of proportionality.^23^ This states that the amount of harm enacted should be proportional to the amount of good and, where possible, harm should be minimised (as per McIntyre’s fifth condition of the DDE).^5^

In contrast, RTS is commonly argued to be inconsistent with the DDE. That is because in such cases (but not in gradual cases), doctors are believed to intend to remove consciousness.^20^ This argument has often received strong support^24–26^; where the argument is accepted, RTS is rejected under the DDE.

However, this argument oversimplifies intention. Cellarius and Henry have argued that RTS can be administered with the intention of relief of suffering.^24^ Furthermore, it is likely that doctors intend to render the patient unconscious in at least some cases of GTS.^27^ First, most cases of GTS result in unconsciousness.^27^ Second, unconsciousness and relief of suffering lie along a causal pathway.^27^ As Anscombe and later Sulmasy have argued, where an actor seeks an outcome (relief of suffering) but comprehends that a separate event (unconsciousness) is required for the outcome to be achieved, then the actor cannot plausibly deny intending this separate event.^28 29^

These criticisms cast doubt over claims that the distinction between RTS and GTS is ethically significant. Intentions likely overlap between these practices. Further, even where doctors pursue unconsciousness, this is not always a ‘bad’.^8^ If consciousness just consists of extremely negative experiences, there can be no value to it. Indeed, it can be a disvalue. Ultimately, RTS cannot be rejected on the basis of intention alone.

We will therefore focus the discussion on the quality of symptom relief provided by the two practices. The problem with titrated (gradual) sedation is that it subjects the patient to intermittent suffering. By definition, doctors wait for signs of symptom recurrence to administer additional doses of sedatives. Savulescu has highlighted the possibility of ‘undershoot(ing)’ during this process and subjecting the patient to a level of suffering that is unnecessary.^30^

Furthermore, empirical studies suggest that sedated patients can still experience suffering despite appearing comfortable to the doctor, especially where the level of sedation is light, as is endorsed by GTS.^31 32^ If lightly sedated patients cannot communicate their suffering, this brings into question the ability of doctors to recognise signs of symptom recurrence.^8^ As a result, GTS might sedate a patient to a level that appears to relieve suffering but does not. In these cases, the patient might suffer, possibly significantly, in the last period of their life. While this risk might also apply to RTS, the risk of inadequate and uncommunicated suffering is relatively less likely in cases of rapid sedation to deep unconsciousness.

### Imminently dying

Finally, TS is commonly described as ‘sedation of the imminently dying’.^33^ Although vague, ‘imminent death’ is generally defined as death expected to occur within hours to days.^33 34^ According to Cellarius, the ‘imminence condition’ has received widespread acceptance in the literature, despite lacking explicit justification.^35^ Intuitively, it may be based on the concern to avoid a ‘premature death’. However, Cellarius argues that where a dying patient’s level of suffering is high, an earlier death is not necessarily a ‘bad’ for the patient.^35^ Importantly, even where an earlier death involves the intentional hastening of death, it can be deemed permissible in this setting as part of the right to relief of suffering. Furthermore, part of our reason for focussing this paper on jurisdictions that permit assisted dying is that in these places it is not seen as necessarily all things impermissible to hasten death (even though that would unquestionably be contrary to the DDE).

The imminence condition denies some patients with refractory symptoms (like Mrs Johnson) access to TS as a means to alleviate their suffering. It is sometimes assumed that suffering is refractory only when death is imminent.^36^ However, refractory symptoms can arise earlier in the dying process. Take, for example, an individual with motor neuron disease who, over the course of several months to years, progressively loses their capacity to speak, then to swallow (requiring a nasogastric tube), and then to breath (requiring respiratory support). This patient may develop intense distress during this process, yet they are denied access to TS until such a time as they are close to death.

It is important at this point to introduce the discussion of artificial nutrition and hydration (ANH). TS is commonly provided without the administration of ANH.^14^ In cases of imminent death, patients are unlikely to be naturally eating and drinking, and consequently omitting ANH is unlikely to hasten death.^5^ If the patient has greater than 2 weeks to live and hydration and nutrition are stopped, it is likely that death will occur sooner (as a result of starvation or dehydration) than it otherwise would.^37^ Subsequently, most guidelines draw the line for administering TS at no earlier than 2 weeks prior to the patient’s expected time of death.^1 38 39^

There are several problems with the 2-week limit. First, patients with greater than 2 weeks to live who experience intolerable or refractory suffering are denied access to ETS, even where they understand and accept the risk of a hastened death (eg, the case of Mrs Johnson). Second, even if omitting ANH were to hasten death, it is permissible to withhold it.^40^ ANH is classified as a medical treatment which may be refused, withheld, or withdrawn if deemed medically futile, or at the request of an informed and competent patient.^41 42^ Rather than removing the option, ANH might be offered in cases of TS in patients who are not imminently dying. Then the patient could make an informed decision about whether or not they wish for TS without ANH to be provided. Even where the clinician is aware that the administration of TS without ANH will likely hasten death, patients should be entitled to make this decision as part of their right to relief of suffering at the end of life.

ETS performed in these instances might be viewed as a form of intentionally ending life (especially where the patient has greater than 2 weeks to live and where ANH is withheld). However, it is not necessarily the case that the clinician intends death in these cases. As an analogy, consider the example of a doctor withdrawing ventilation from a quadriplegic patient who no longer wishes to live. Although the doctor knows that they are thereby hastening death, that is not necessarily their intention, and (potentially regardless of their intention) it is permissible and perhaps ethically obligatory to do so. A qualitative study of palliative care physicians found that the intention-foresight distinction does in fact matter to those administering sedation at the end of life, especially in cases of earlier TS and TS in patients with more difficult to control symptoms.^43^ Furthermore, even if the clinician does intend death, it is not impermissible to withhold or withdraw a treatment that a patient is refusing. Importantly, in this paper, we have focused on jurisdictions where assisted dying is legal, and thus where the intentional ending of life is deemed permissible in some circumstances.

### Interim conclusion

In this section, we have challenged the standard limits placed on TS as outlined by de Graeff and Dean—specifically the requirements for (1) refractory symptoms, (2) gradual sedation and (3) imminent death. In all three cases, it is not clear that the arguments for the limits clearly outweigh the arguments against. There is a reliance on the DDE to support these limits, but intention is not always clear-cut and is insufficient to distinguish acceptable from unacceptable sedation. A consideration of other ethical principles is required. An overly cautious application of TS might prolong suffering. Furthermore, such restrictions potentially conflict with patient autonomy and the importance of a patient’s own judgement about when suffering becomes intolerable and when sedation might be desirable, and their own conception of a good death. At least in some circumstances, ETS is morally permissible.

## In what circumstances is ETS morally permissible?

In this section, we will explore the circumstances in which ETS is morally permissible, seeking to define a new set of eligibility criteria for access to sedation at the end of life.

### Non-refractory suffering

As with other treatment options for suffering, access to ETS should be reserved for patients with an appropriate clinical indication, that is a diagnosable medical condition, and then guided by the level and nature of the patient’s suffering. The appropriateness of an intervention to manage suffering should be weighed against the risk profile of this intervention. If another treatment option is likely to effectively manage the patient’s current level of suffering and is associated with fewer risks than ETS, it should be offered first. This is the standard approach taken to analgesia. If a doctor believes a patient’s pain could be reasonably controlled with simple analgesia, that would ordinarily be trialled first.

Importantly, however, this does not mean that ETS should be reserved as a therapy of last resort. ETS might be appropriate in response to non-refractory suffering where the patient’s level of suffering is great and other options (such as increasing doses of opioids) are likely to fail. Opioids commonly fail to comprehensively manage suffering at the end of life (approximately 12%–18% of dying patients worldwide receive TS under current policies).^2^ Again, this is not fundamentally different from approaches taken to pain relief. In cases of severe pain, simple analgesia may be bypassed in lieu of earlier access to opioids. Where opioids are likely to fail, sedation may be employed. There would be value in more empirical research to help identify when earlier treatment options are likely to fail at the end of life (ie, to identify any clinical commonalities between the patients in whom opioids fail). In these circumstances, sedation is the next best treatment option and access to ETS would respect a right to relief of suffering.

### Rapid sedation

The principle of proportionality means that the potential for relief of suffering (a ‘good’ outcome) should be proportionate to the risks associated with TS (‘bad’ outcomes).^23^ RTS achieves high-quality relief of suffering through both rapid sedation (no intermittent suffering through titration) and deep sedation (minimal risk of residual suffering). It is sometimes assumed that this is necessarily disproportionate and hence impermissible at the end of life.^23^ However, this is not always the case.

First, cases will vary. Some patients can be stabilised on small doses of sedatives.^31^ However, in other patients, where their level of suffering is great, the lowest doses of sedatives required to achieve adequate relief of suffering might in fact be large doses of sedatives which rapidly sedate to unconsciousness. In these circumstances, RTS would be proportionate (and is morally permissible).

Second, the judgement about what would be proportionate is value based, not technical. Some would regard the risks of RTS as proportionate, others would not. Given the risk of inadequate relief of suffering with GTS, if a doctor is unsure if GTS is likely to be effective, the patient (or their proxy) should be able to choose between GTS and RTS. (see below for more detailed discussion of the application of ETS to patients who lack decision-making capacity, eg, as a consequence of end-of-life distress.) Empirical data suggest that patients are interested in accessing RTS at the end of life. In a 2021 survey of the UK public, 88% of respondents expressed a desire to be offered terminal anaesthesia (a form of RTS) in the dying phase.^44^

Next, RTS might be dismissed because of concerns about the intent of the physician. Doctors are commonly said to intend to render the patient unconscious only in cases where they rapidly sedate the patient.^20^ However, as discussed earlier, there is overlap in the intentions between cases of gradual and rapid sedation. Furthermore, there are some situations at the end of life in which unconsciousness might be desirable. First, to sedate to unconsciousness is to eliminate suffering through awareness.^32^ If unconsciousness provides the mechanism through which suffering is controlled in these cases (and there is no better alternative), then unconsciousness is arguably a desirable, and intended, outcome. Second, Takla *et al* argue that some experiences might render mere consciousness a ‘bad’, or at least no longer a ‘good’.^8^ These experiences may include pain, nausea, dyspnoea, delirium, or existential distress in the terminally ill. Janssens *et al* argue that TS is defined by the intended removal of consciousness, which is justified in ‘grave’ circumstances at the end of life.^10^ We posit that such grave circumstances should include anything that poses a significant compromise to the quality of one’s consciousness at the end of life.

**Figure 1:**
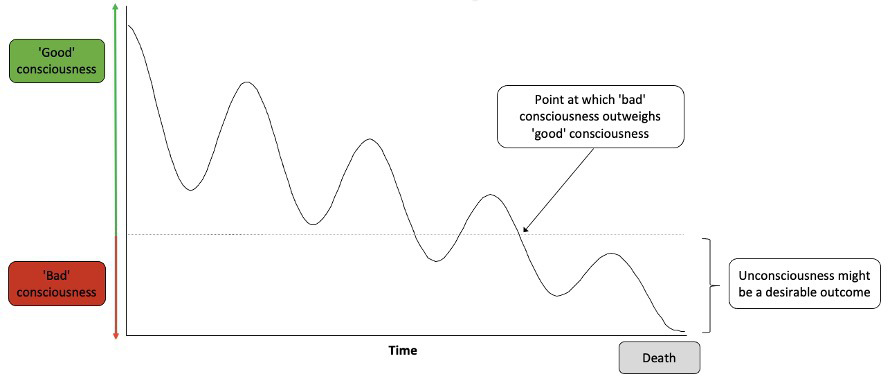
Quality of consciousness at the end of life.

Many patients (like Mrs Johnson) experience existential symptoms at the end of life, including a fear of dying, helplessness, hopelessness, meaninglessness, loss of dignity, loss of sense of self, regret and discontentment.^45^ Compared with physical symptoms, existential symptoms are often harder to recognise, measure and treat.^45^ A patient is less likely to meaningfully participate in their conscious existence where they are questioning the contents, meaning and longevity of that very existence. Existential symptoms can range in severity and can even come and go. However, where they constitute a significant compromise to the quality of one’s consciousness, and particularly where the trajectory of distress is such that negative future experiences are likely to dominate (as per Mrs Johnson’s case), reductions in consciousness might be desirable (or at least morally neutral). The desirability of unconsciousness in cases of ‘bad’ consciousness is depicted in figure 1.

In summary, RTS is proportionate, and therefore, morally permissible, in the following circumstances: (1) the patient’s level of suffering is high and GTS is likely to be ineffective and (2) consciousness is reasonably deemed by the patient to be a ‘bad’ (or at least not a ‘good’) and unconsciousness can therefore be aimed at through RTS.

### TS versus assisted dying

In this section, we will compare TS to assisted dying in jurisdictions where the latter is legal. We will use the Australian state of Victoria as an example. In jurisdictions in which assisted dying is legal, ETS should at least be permitted when it meets similar criteria. (Of course, this leaves open whether it should be permitted in a wider range of situations or in jurisdictions in which assisted dying is illegal.)

Victoria introduced legislation to permit assisted dying in 2017. The Voluntary Assisted Dying (VAD) Act allows people to request access to VAD where they meet all of the following criteria^46^:

1. They have an incurable medical condition.
2. They are experiencing intolerable suffering.
3. The medical condition is expected to cause death within 6 months (or 12 months if the person has a neurodegenerative condition).
4. They have decision-making capacity.
5. They are 18 years or older.

In contrast, there is no specific legislation governing TS in Victoria, which is instead guided by professional guidelines.^47^ TS is described in these guidelines as an acceptable therapy for the treatment of suffering at the end of life.^47^ TS can be considered if the following criteria are met^47^:

1. The patient has an advanced or terminal medical condition.
2. Death is imminent (estimated life expectancy of 2 weeks or less).
3. Symptoms (physical or non-physical) are refractory, meaning that they are “irreversible” and all other treatment options have failed.
4. The patient (or advance care directive or proxy) consents to the therapy.
5. Sedatives are administered proportionally (‘minimum dose of sedatives needed to achieve acceptable relief of suffering’).

Given that TS currently exists in a relatively unregulated space, it is likely that ETS already occurs to some degree on a case-by-case basis.^43^ If this is true, then arguably there is an even greater need to define a clear set of criteria for performing TS outside of the traditional limits. Doing so would provide patients with clearer pathways to access an intervention which they are already seeking and would enable all patients who want TS to be able to access it.

Victorian guidelines place significant emphasis on the distinction between a ‘therapy’ to treat suffering and a ‘process’ to control the timing of one’s own death in response to suffering at the end of life (VAD).^46 47^ Table 1 lists and compares the current eligibility criteria for these two practices in the State of Victoria.

**Table 1:** Comparison of the current eligibility criteria for TS and VAD in Victoria, Australia

	TS	VAD
Who	Terminal medical condition	Incurable medical condition
Age	Any age	18 years or older
Estimated life expectancy	2 weeks or less	6 months or less (or 12 months if neurodegenerative condition)
Suffering	Refractory	Intolerable
Consent	Patient or advance care directive or proxy	Individual initiates request, assessed by two separate medical practitioners to have decision-making capacity, enduring request for 10 days

TS, terminal sedation; VAD, voluntary assisted dying.

Existing criteria for TS are in some domains more restrictive, and in others more permissive than those that apply for VAD. The distinction between TS and assisted dying has been discussed previously.^3 9 48^ However, expanding the criteria for TS potentially blurs this distinction, especially where ETS foreseeably hastens death. Some will see this as a reason to avoid extending the limits on TS. However, if ETS is permitted, there remain some fundamental differences from VAD which can help justify their moral and/or legal distinction:

- The two practices use different medications. Barbiturates and other sedatives such as midazolam have different mechanisms of action and achieve different effects. Some patients will be interested in the difference between these medications; these preferences are morally relevant as per a right to relief of suffering and will be discussed further below.
- The two practices are administered to achieve different effects (and thus vary in ‘intention’). VAD is carried out to swiftly bring about death. Sedatives are administered to remove consciousness (where this consciousness is associated with suffering).
- ETS does not necessarily hasten death. The life shortening effects of ETS will vary depending on the individual patient and the doses of sedatives employed. The hastening of death is therefore not a fundamental component of ETS, whereas it is clearly central to VAD. Where ETS foreseeably hastens death, patient preferences and the DDE are helpful to morally differentiate ETS from VAD.
- Unconsciousness until the point of death is not the same as death. First, patients in an irreversible coma are managed entirely differently to patients who have died. Second, unconsciousness induced by sedatives is at least theoretically reversible up to a point; whereas assisted dying is by definition irreversible.

### Greater than 2 weeks to live

One reason to restrict the time period for accessing TS is based on beliefs about the timing of suffering. Some appear to assume that suffering only ever becomes intolerable or refractory when death is imminent.^36^ However, as discussed above, some slowly progressive diseases may cause intense distress and suffering for a considerable period before they lead to death.

A more plausible basis for the imminence condition is based on a desire to avoid hastening death significantly. Of course, very large doses of sedatives could be lethal at any stage of the dying process, including within the 2-week limit.^8^ It is reasonable to assume that the appropriate administration of sedatives, whether or not paired with ANH, will not hasten death significantly for a patient with such a short remaining period of life.^37^ But TS for patients with more than 2 weeks to live (particularly where ANH is also withheld), will clearly hasten death in at least some cases. However, at least in jurisdictions where assisted dying is permissible, the risk of hastening death is not an absolute impediment to end of life practices. It will be useful to distinguish these two categories.

#### Patients who meet the eligibility criteria for assisted dying

In jurisdictions where assisted dying is legal, there are strict eligibility criteria for access to assisted dying which set out the situations in which hastening death might be permissible. It would be consistent for ETS to be accessible in patients with greater than 2 weeks to live where the same eligibility criteria for assisted dying are met. Ideally, in such jurisdictions, ETS should be governed with similar legal oversight and a clear set of eligibility criteria.

Using Victoria as an example, there are strong ethical grounds for allowing an individual to request access to ETS where they: (1) have an incurable medical condition, (2) are experiencing intolerable suffering, (3) have 6 months or less to live, (4) have decision-making capacity and (5) are 18 years or older. Individuals in these circumstances should be able to choose between VAD and ETS. This is, at the very least, a logical extension of the arguments in favour of access to assisted dying in the same instances. Mrs Johnson (described earlier in the clinical case) meets all the eligibility criteria for VAD in Victoria but is ineligible for TS. Accordingly, if she were a resident in Victoria, she would be granted access to ETS. Of course, in such circumstances, many may choose VAD over ETS, but not necessarily all. There are some individuals who are opposed to euthanasia but who would accept sedation at the end of life.^44^

#### Patients who do not meet the eligibility criteria for assisted dying

We now consider whether there any circumstances in which it is morally permissible for a patient to access ETS beyond the 2-week limit where the eligibility criteria for assisted dying are not met.

First, assisted dying is restricted in many jurisdictions to those who are terminally ill and suffering. Both of these criteria would ordinarily apply to patients seeking ETS as a treatment option for suffering at the end of life. The role for ETS in individuals who do not meet these criteria (ie, are not at the end of life) is beyond the scope of this paper.

Second, assisted dying is further restricted in many jurisdictions to those who are able to explicitly and reliably indicate their wishes. For consistency, one approach to ETS (in patients with more than 2 weeks to live) would be to similarly limit it to those who (4) have decision-making capacity and (5) are 18 years or older. However, those are not standard criteria for ethically justifiable TS. Patients who lack decision-making capacity at the end of life are no less likely to experience intolerable or refractory suffering. Indeed, some forms of suffering may directly contribute to (or result from) decreases in capacity—for example, delirium, agitation, drowsiness or cognitive decline.

Therefore, a second approach would be that if the doctor deems sedation to be a clinically appropriate intervention to relieve suffering and sedation is in accordance with the patient’s values (as determined by an advance care directive or next of kin), there is good reason for ETS to be administered to a patient who lacks decision-making capacity, even if they have greater than 2 weeks to live.

Access to ETS in these circumstances can be supported by both a right to relief of suffering and the DDE. Dying patients who lack decision-making capacity still retain their right to relief of suffering. Further, ETS might be morally permissible if it is administered with the intention of relieving suffering and the potential for hastening death is merely foreseen. Importantly, the DDE could still be applicable in cases where a hastened death is all but guaranteed (Sulmasy gives the analogy of a doctor foreseeing, but not intending, hair loss during chemotherapy).^28^

**Figure 2:**
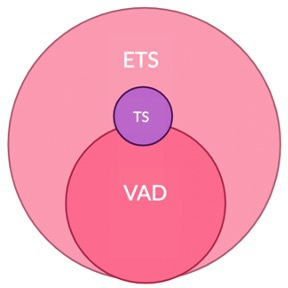
Convergence between the eligibility criteria for VAD, ETS and TS in Victoria. ETS, expanded terminal sedation; TS, terminal sedation; VAD, voluntary assisted dying.

The potential convergence between the criteria for VAD, ETS and TS in Victoria is depicted in figure 2.

VAD and ETS overlap where all the eligibility criteria for VAD are met. ETS can be distinguished from VAD where the criteria for decision-making capacity and/or 18 years or older are not met. While, at their core, TS and ETS are the same practice, the pool of eligible patients is bigger in ETS. TS and VAD overlap where an individual who meets the criteria for TS makes a request for VAD (although this would be very few patients given the short duration of survival anticipated and the challenge of accessing VAD urgently).

### Summary and recommendations

We have described (and argued for) some of the circumstances in which it is morally permissible for TS to be expanded beyond its limits. We propose the following set of eligibility criteria for ETS:

1. The patient has an incurable medical condition.
2. The patient is experiencing suffering, which is either:
  - Refractory (all possible treatment has failed). OR
  - Intolerable (and further treatment options are likely to fail).
3. The patient has an estimated life expectancy of 6 months or less.
4. The patient (or advance care directive or proxy) consents to the therapy.
5. Sedatives are administered:
  - Gradually. OR
  - Rapidly (if gradual sedation is likely to be ineffective or if unconsciousness is a clinically desirable outcome).

We have described a set of criteria for Victoria, Australia, but these eligibility criteria could be transferrable to other jurisdictions where assisted dying is legal. At the present time, TS criteria typically remain restrictive in such countries, upholding the 2-week limit.^8^ Access to ETS could be adjusted to mirror the assisted dying criteria in each jurisdiction. Most jurisdictions uphold the requirement for a medical condition associated with unbearable suffering.^49^ Unlike Victoria, other jurisdictions do not necessarily impose a strict life expectancy window for access to assisted dying. Consequently, in these jurisdictions ETS need not be limited to patients with less than 6 months to live. Some jurisdictions, including the Netherlands and Belgium, have removed the requirement for decision-making capacity, allowing patients to access assisted dying through an advance care directive.^49^ As such, in these jurisdictions ETS might be accessed in the same instances.

Finally, while not the central focus of this paper, these criteria could also be applied to jurisdictions where assisted dying is illegal, where there is an equal, if not greater, need for access to ETS. As outlined above, there are key conceptual differences between assisted dying and ETS which might render ETS permissible even where assisted dying is not. As a minimum, dying patients with more than 2 weeks to live should be able to access ETS where it is an appropriate treatment option for their suffering, irrespective of the legal status of assisted dying. More research is needed to examine the moral permissibility of ETS for patients with longer periods of predicted survival in jurisdictions where assisted dying is illegal. One option in such countries may be the combination of competent refusal of food and fluids along with the provision of palliative care (also known as voluntary palliated starvation), possibly including ETS.^50^

## Objections

Some of the above suggestions for expanding TS will be controversial. For example, some may wish to limit sedation to a gradual form and to cases of refractory suffering because of the possibility that suffering could be relieved by alternative methods, or by lower doses. Others may be opposed to the expansion of TS where this foreseeably hastens death. ETS itself could also be associated with new medical challenges, such as pressure ulcers related to the longer duration of sedation as well as the increased demand on medical beds. Patients should be aware of these risks, offered the option of further analgesics/gradual sedation and be supported in this choice if they wish. However, for the reasons outlined above, patients who do not share this view and wish for earlier or more rapid sedation should also be supported. This is especially true in jurisdictions where methods of hastening death are already permissible.

Others may regard consciousness as always valuable, perhaps reflecting on the positive meaning and experience that patients can find in their dying phase. Such individuals may wish to avoid deliberate or deep sedation and are of course entitled to that view and to request it in their own care. However, patients who do not share that view, and who do not wish to remain conscious at the end of life should also be supported.

If ETS is made available to patients who lack capacity, there may be concerns about abuse or bias in decision-making. Furthermore, some patients might receive ETS where, if they had decision-making capacity, they would not have consented to this practice. However, this risk applies to all medical decisions made in patients who lack decision-making capacity, including decisions about withdrawal of life sustaining treatment and current forms of TS. We do not think that the possibility of abuse means that life-sustaining treatment should never be withdrawn from patients who lack capacity (even if the patient would be likely to survive for more than 2 weeks if treatment were continued), or that TS must be restricted to those with capacity. On the contrary, the appropriate response is to ensure that decisions are made carefully and rigorously in accordance with the patient’s best interests.

Procedural safeguards can be employed to regulate the practice of ETS and minimise these risks. Given the potential use of ETS in patients who are unable to make decisions for themselves, these safeguards will differ from assisted dying safeguards, which are often aimed at ensuring decision-making capacity. Furthermore, the safeguards will be modified to reflect the conceptual differences between ETS and assisted dying, outlined above. Potential safeguards for ETS could include:

- The patient should be reviewed by two separate medical practitioners who independently agree that sedation is clinically appropriate in the management of the patient’s suffering. A formal multidisciplinary best interests meeting or review by a clinical ethics committee might be invoked in some or all cases.
- Where possible, the medical team should discuss in advance the patient’s preferences about access to sedation during the dying phase. Patients should be encouraged to make an advance directive detailing these preferences.
- The patient’s family should be involved in discussions about the patient’s views and best interests as they relate to sedation during the dying phase. Where there is disagreement between the family and the clinical team about the patient’s best interests an application to the court might be made.
- An opt-out register can be created for patients who do not wish to receive ETS, including if they lose decision-making capacity. A preference to not receive ETS can be communicated at any stage that the dying patient has capacity and then formalised in an advance directive (as per any other preference concerning end-of-life care).

## Conclusion

The relief of suffering is a fundamental goal of end-of-life care. However, despite the technical knowledge and ability to pharmacologically relieve suffering in dying patients, a significant proportion of dying patients continue to suffer. One reason for this is that a number of dying patients remain ineligible for sedation due to the traditional limits placed on its use.

In this paper, we have explored the moral permissibility of expanding TS beyond those limits, focussing on jurisdictions where assisted dying is legal. The legal option of assisted dying does not remove the need for treatment of suffering at the end of life. ETS provides a potential means of relief of suffering for patients who are not eligible for assisted dying. Furthermore, it may be important for some patients to be able to choose between these options.

We have defended the option of ETS in a set of clearly defined circumstances. Future research is needed to clarify the circumstances in which opioids and/or gradual sedation are likely to be ineffective, the practicalities of providing ETS to dying patients who lack decision-making capacity, and the potential boundaries of ETS in jurisdictions where assisted dying remains illegal.

There can be good reasons to limit the use of certain medicines—for example, where they would do more harm than good, or where they would be an unreasonable use of resources. However, the current limits placed on sedation in dying patients are not justified. They should be expanded.

## Data Availability

No data are available.

## Footnotes

Fictional case.

While end-of-life care already includes a range of (sometimes confusing) terminology, we have elected to use this new term to help differentiate what we are proposing from existing practice and hopefully ignite discussion around potential new eligibility criteria for access to TS. Of course, if the arguments of this paper are accepted, then the new term will no longer be required: ‘ETS’ will become ‘TS’.
